# RNA Interference Analysis Reveals the Positive Regulatory Role of Ferritin in Testis Development in the Oriental River Prawn, *Macrobrachium nipponense*

**DOI:** 10.3389/fphys.2022.805861

**Published:** 2022-02-18

**Authors:** Shubo Jin, Hongtuo Fu, Sufei Jiang, Yiwei Xiong, Hui Qiao, Wenyi Zhang, Yongsheng Gong, Yan Wu

**Affiliations:** Key Laboratory of Freshwater Fisheries and Germplasm Resources Utilization, Ministry of Agriculture, Freshwater Fisheries Research Center, Chinese Academy of Fishery Sciences, Wuxi, China

**Keywords:** *Macrobrachium nipponense*, ferritin peptide, male sexual development, RNAi, testosterone

## Abstract

Ferritin plays an essential role in organismic and cellular iron homeostasis in *Macrobrachium nipponense*. In this study, we aimed to investigate the role of ferritin in the sexual development of male *M*. *nipponense*. According to the qPCR analysis of different tissues and developmental stages, ferritin exhibited high expression levels in the testis and androgenic gland, from post-larval developmental stage 5 (PL5) to PL15, indicating that it may be involved in gonad differentiation and development, especially in male sexual development. *In situ* hybridization and qPCR analysis in various reproductive cycles of the testis indicated that ferritin may play an essential role in spermatogonia development in *M*. *nipponense*. RNAi analysis revealed that ferritin positively affected mRNA expression of the insulin-like androgenic gland (*Mn-IAG*) and the secretion of testosterone, and thus positively affected testis development in *M. nipponense*. This study highlighted the functions of ferritin in the sexual development of male *M. nipponense* and provided important information for the establishment of a technique to regulate the process of testis development in *M. nipponense*.

## Introduction

The oriental river prawn, *Macrobrachium nipponense* (Crustacea; Decapoda; and Palaemonidae), is an important commercial species in China (Cai and Shokita, 2006; Salman et al., 2006; Ma et al., 2011), with an annual production of 225,321 tons in 2019 (Zhang et al., 2020). There are significant differences in growth performance of male and female *M*. *nipponense* in aquaculture systems. Compared to their female counterparts, male *M*. *nipponense* exhibit better growth performance (Ma et al., 2011). Rapid gonad development has negative effects on the sustainable development of the *M. nipponense* aquaculture industry. A previous study reported that the both testis and ovaries of prawns are mature at 40 days after hatching (Jin et al., 2016). This results in inbreeding between the newborn prawns. Inbreeding leads to multiple generations in the same pond and the degradation of germplasm qualities, resulting in the prawns with smaller market size, and decreased ability to resist diseases (Fu et al., 2012; Jin et al., 2021a,b). Therefore, studies on the sexual development of male *M. nipponense* have received increased attention in recent years, with the aim of establishing an artificial technique to produce all male progeny on a commercial scale and regulate the process of testis development.

The androgenic gland has become a target tissue for studying male sexual differentiation and development in crustacean species in recent years. The hormones secreted by the androgenic gland have been reported to play positive regulatory roles in the processes driving male sexual differentiation and characteristics, in particular, the promotion of testes development in crustacean species (Sagi et al., 1986, 1990). Insulin-like androgenic gland hormone (*IAG*) is the main gene expressed and analyzed in the androgenic gland (Ventura et al., 2009, 2011; Rosen et al., 2010). It has been shown to play essential roles in promoting male sex determination and sex differentiation in most crustacean species, including *Fenneropenaeus chinensis* (Li et al., 2012), *Scylla paramamosain* (Huang et al., 2014), *Lysmata vittata* (Liu et al., 2021), *Fenneropenaeus merguiensis* (Zhou et al., 2021), and *M. nipponense* (Li et al., 2015a). Silencing of *IAG* in male *M. rosenbergii* by RNA interference (RNAi) may also lead to a complete sex reversal (Ventura et al., 2012). A previous study reported that *IAG* was expressed in the androgenic gland in *M. nipponense* (Ma et al., 2016). Thus, the genes in the androgenic gland have received a lot of attention in recent years and the transcriptome and miRNA library of the androgenic gland have been constructed for *M*. *nipponense* (Jin et al., 2013, 2015). A series of genes identified in the androgenic gland transcriptome have been analyzed and proven to be involved in the sexual development mechanisms of male *M*. *nipponense* (Jin et al., 2014, 2018b; Li et al., 2015a; Ma et al., 2016).

Previous studies have found that ferritin regulates cellular and organism-wide iron homeostasis, and thus protects cells from damage by excess iron (Picard et al., 1996; Konijn et al., 1999; Kakhlon et al., 2001; Torti and Torti, 2002). In addition, ferritin has multiple other functions, e.g., in development, cell activation, and angiogenesis (Parthasarathy et al., 2002; Coffman et al., 2008; Alkhateeb and Connor, 2010; Wang et al., 2010). A total of three ferritin subunits were identified in *M. nipponense*, including ferritin, ferritin light-chain subunit, and ferritin heavy-chain subunit. A previous study revealed that ferritin with accession no. KC825355 in GenBank plays an essential role in organismic and cellular iron homeostasis in *M. nipponense* (Sun et al., 2014). However, ferritin was also highly expressed in the androgenic gland, which is predicted to have additional roles in the mechanisms of sexual development in male *M*. *nipponense* (Jin et al., 2018a).

In this study, we aimed to further analyze the function of ferritin in *M*. *nipponense*, especially in relation to its role in male sexual development, using Quantitative real-time PCR (qPCR), *in situ* hybridization, and RNAi, combined with histological observations and testosterone measurements. The results of this study highlighted the functions of ferritin in *M*. *nipponense*, providing a basis for further studies on the mechanism of male sexual development in other crustacean species.

## Materials and Methods

### Ethics Statement

Permission for all experiments involving *M. nipponense* was obtained from the Institutional Animal Care and Use Ethics Committee of the Freshwater Fisheries Research Center, Chinese Academy of Fishery Sciences (Wuxi, China).

### The qPCR Analysis

The relative mRNA expression of *Mn-ferritin* was measured using qPCR. Different mature tissues included the testis, androgenic gland, ovary, intestine, hepatopancreas, and heart. The full-sibs population was hatched and cultured, and specimens at different larval and post-larval developmental stages were collected every 5 days during their maturation process. Testes were collected during the reproductive season in July, when the water temperature was ≥28°C and the illumination time was ≥16 h. Testes were also collected during the non-reproductive season in January, when the water temperature was ≤15°C and illumination time was ≤12 h. Samples of each tissue or stage were collected from fifty individual prawns. Ten prawns were pooled together to form one biological replicate, in order to minimize the effects of individual differences and five biological replicates were conducted. The procedures of RNA isolation and synthesizing cDNA have been described in detail in previous studies (Jin et al., 2014, 2018b). Briefly, total RNA was extracted from each tissue, using the UNlQ-10 Column TRIzol Total RNA Isolation Kit (Sangon, Shanghai, China) following the manufacturer’s protocol. A total of 1 μg total RNA from each tissue was used to synthesize the cDNA template by using the PrimeScript RT reagent Kit (Takara Bio Inc., Japan). The expression level of each tissue was determined using the UltraSYBR Mixture (CWBIO, Beijing, China). All of the qPCR analyses in this study were performed on the Bio-Rad iCycler iQ5 Real-Time PCR System (Bio-Rad, Hercules, CA, United States), which was used to carry out the SYBR Green RT-qPCR assay. All qPCR reactions were run in three technical replicates. Table 1 lists the primers used for qPCR analysis, including the eukaryotic translation initiation factor 5A (EIF 5A), which was identified as a suitable reference gene for PCR analysis in *M. nipponense* (Hu et al., 2018). The relative mRNA expressions of *Mn-ferritin* were calculated, based on the 2^–ΔΔCT^ comparative CT method (Livak and Schmittgen, 2001).

**TABLE 1:** Universal and specific primers used in this study.

Primer name	Nucleotide sequence (5′→3′)	Purpose	GenBank
Fer-RTF	CCGAAATCCGCCAGAACTAC	FWD primer for ferritin expression	KC825355
Fer-RTR	GCTTATCGGCATGCTCTCTC	RVS primer for ferritin expression	
Fer anti-sense probe	GCTGGCATACAATTCCATGTTGATCTGCTTGTTAATG	Probe for ferritin ISH analysis	
Fer sense probe	CATTAACAAGCAGATCAACATGGAATTGTATGCCAGC	Probe for ferritin ISH analysis	
Fer RNAi-F	TAATACGACTCACTATAGGGTGCTCTTCCTGGTATGTCCC	FWD primer for RNAi analysis	
Fer RNAi-R	TAATACGACTCACTATAGGGCCAAGCTCCTTGATGGACTC	RVS primer for RNAi analysis	
EIF-F	CATGGATGTACCTGTGGTGAAAC	FWD primer for EIF expression	MH540106
EIF-R	CTGTCAGCAGAAGGTCCTCATTA	RVS primer for EIF expression	

### *In situ* Hybridization

The mRNA locations of *Mn-ferritin* were analyzed by *in situ* hybridization. Paraformaldehyde (4%) (Sangon, Shanghai, China) was used to fix the tissue samples until the experiment was carried out. The androgenic gland and hepatopancreas were sampled during the reproductive season (28°C), while testes were collected from both of the reproductive season (28°C) and non-reproductive season (15°C). Procedures of primer design and *in situ* hybridization have been described in detail in previous studies (Jin et al., 2018b; Li et al., 2018). Briefly, Primer 5 software was used to design the anti-sense and sense probes with a DIG tag for the *in-situ* hybridization study, based on the cDNA sequence of *Mn-ferritin*. Table 1 lists the primers used for *in situ* hybridization analysis, and the primers with DIG signals were synthesized by Shanghai Sangon Biotech Company. The anti-sense probe and sense probe were prepared for the experimental group and the control group, respectively. All of the collected testes (15 and 28°C), androgenic glands (28°C) and hepatopancreas (28°C) were embedded by paraffin. The *in situ* hybridization study was performed on 4 μm thick sections of paraffin-embedded tissues using the ZytoFast PLUS CISH implementation kit (ZytoVision GmBH, Bremerhaven, Germany), following the manufacturer’s protocol. The sections were incubated in 3% H_2_O_2_ for 10 min. After rinsing in deionized water (DW), target retrieval was achieved using 0.5 mg/ml pepsin digestion in a humidity chamber for 10 min. The slides were incubated in EDTA solution at 95°C for 15 min after being washed in DW. The slides were then washed in DW, drained off, and 20 μL of CISH anti-sense probe and sense probe were poured over each slide. Denaturation was carried out at 75°C for 5 min, followed by hybridization at 37°C for 60 min in the ThermoBrite TM hybridization chamber (Vysis Inc, Downers Grove, IL, United States). Tris-buffered-saline (TBS) washing was carried out at 55°C for 5 min, and then at room temperature for five min. Mouse-anti-DIG (ZytoVision GmBH, Bremerhaven, Germany) was dropped over each slide, and incubated in a humidity chamber at 37°C for 30 min. Three washings were carried out with TBS, each lasting for 1 min, before and after incubating slides in anti-mouse-HRP-polymer for 30 min at room temperature. The 3,3′-diaminobenzidine (DAB) solution was prepared as per guidelines (ZytoFast PLUS CISH; ZytoVision GmbH) and 50 μL was poured over each slide for 10 min at room temperature. After washing, hematoxylin–eosin was used for counterstaining (see below for details). Slides were dehydrated in graded ethanol solutions, air dried and mounted with a mixture of distyrene, plasticizer, and xylene (DPX). Slides were examined under a light microscope.

### RNA Interference Analysis

RNA interference was performed to analyze the novel regulatory roles of ferritin in the mechanism of sexual development in male *M*. *nipponense*. A specific RNAi primer with a T7 promoter site was designed in the open reading frame of *Mn-ferritin*, using Snap Dragon tools^1^ (Table 1). The *Mn-ferritin* dsRNA was synthesized using the Transcript Aid*™* T7 High Yield Transcription kit (Fermentas Inc., Burlington, ON, Canada), based on the manufacturer’s protocol. Six hundred healthy, mature, male *M*. *nipponense* with a bodyweight of 2.11–2.78 g were collected approximately 5 months after hatching from Tai Lake in Wuxi, China (120°13′44″E, 31°28′22″N). These male prawns were randomly divided into the RNAi group and 0.9% saline group with each group containing 300 prawns; 0.9% saline group was considered as the negative control. As described in a previous study (Jiang et al., 2014; Li et al., 2018), prawns from the RNAi group were injected with 4 μg/g *Mn-ferritin* dsRNA. Prawns from the control group were injected with an equal volume of 0.9% saline, according to the prawn’s body weight. Androgenic gland samples were collected from both the control group and the RNAi group on days 1, 7, and 14, after 0.9% saline and *Mn-ferritin* dsRNA injection, and the *Mn-ferritin* mRNA expression was measured by qPCR, permitting confirmation of silencing efficiency. The androgenic gland samples were collected from five individual prawns and pooled together to form a biological replicate, and five biological replicates were performed. The mRNA expression of *Mn-IAG* was measured using the same cDNA templates in order to analyze the regulatory relationship between *Mn-ferritin* and *Mn-IAG*.

### Testosterone Measurement

Testes were collected from the both control group and the RNAi group 1, 7, and 14 days after the injection of 0.9% saline and *Mn-ferritin* dsRNA, and stored at −20°C. Testes were collected from forty individual prawns at each time point in both control and RNAi groups. Eight prawns were pooled together to form one biological replicate, in order to produce a total tissue sample weight of 0.2 g, and five biological replicates were performed. The testosterone was extracted from the testes by adding 5 mL 100% methyl alcohol and fully grinding the sample. The ground samples were kept at 4°C for 4 h and centrifuged at 3,000 rpm for 5 min. The supernatant was collected and directly used to measure the content of testosterone. The testosterone concentration in triplicate samples was measured using an Access 2 Immunoassay System (Beckman Coulter, Inc., Brea, CA, United States) (Jin et al., 2019).

### Hematoxylin and Eosin Staining

Hematoxylin and eosin staining was used to measure the morphological differences of the testis between the control group and RNAi group. The procedure of HE staining has been well described in previous studies (ShangGuan et al., 1991; Ma et al., 2006). Briefly, the tissues were first dehydrated by using 50, 70, 80, 95, and 100% ethanol. The tissues were then made transparent and embedded using alcohol: xylene (1:1), xylene, xylene: wax (1:1), and wax. The embedded tissues were sectioned to a thickness of 5 μm using a slicer (Leica, Wetzlar, Germany), and placed on a slide. The slides were then stained with HE for 3–8 min. The slides were observed under an Olympus SZX16 microscope (Olympus Corporation, Tokyo, Japan).

### Statistical Analysis

SPSS Statistics 23.0 (IBM, Armonk, NY, United States) was used to conduct all statistical analyses. The test of homogeneity of variances was performed prior to ANOVA analysis and *t*-test (Sig. >0.05). Meanwhile, a linear regression analysis was performed on each set of data. The mean residual of each group data is close to 0, and the residual deviation is close to 1 for the linear regression analysis, indicating that the residuals of the data are normally distributed and can be analyzed. Statistical differences were identified by ANOVA analysis of variance followed by the least significant difference and Duncan’s multiple range tests. The statistical difference between the control group and the RNAi group on the same day was assessed using the paired *t*-test. Quantitative data were expressed as mean ± standard deviation. A *P*-value < 0.05 was considered to be statistically significant.

## Results

### Expression Analysis in Different Tissues

**FIGURE 1:**
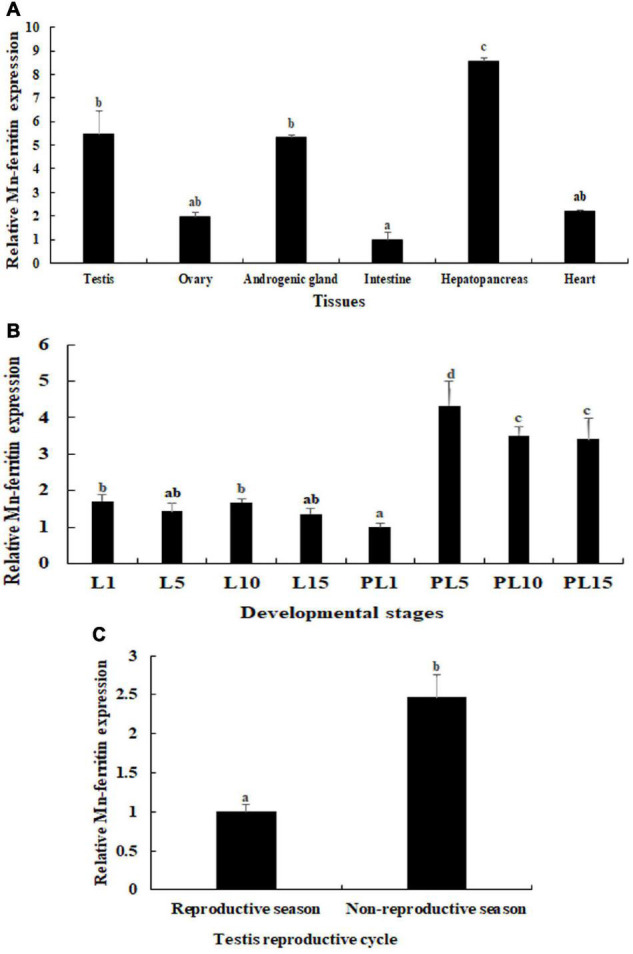
qPCR analysis of *Mn-ferritin* in different tissues and developmental stages. The amount of *Mn-ferritin* mRNA was normalized to the *EIF* transcript level. Data are shown as mean ± SD (standard deviation) of tissues from five biological replicates. Letters indicate expression differences between different samples. Highest expression level of *Mn-ferritin* was observed in the hepatopancreas, followed by the testis and androgenic gland. *Mn-ferritin* expression was generally higher during the post-larval developmental stages than the larval developmental stages, and the expressions remained at a high level from PL5 to PL15. *Mn-ferritin* expression level in the testis of the non-reproductive season was significantly higher than that in the testis of the reproductive season. **(A)** Expression characterization in different tissues. **(B)** Expression characterization in different developmental stages. **(C)** Expression characterization in different reproductive seasons of testis.

The physiological function of *Mn-ferritin* was reflected by tissue distribution in *M*. *nipponense*, verified by qPCR (Figure 1A). According to qPCR analysis, *Mn-ferritin* showed the highest expression level in the hepatopancreas (*P* < 0.05), followed by the testis and androgenic gland. The intestine had the lowest expression level. The mRNA expressions of *Mn-ferritin* in the testis and androgenic gland were over five times higher than that of the intestine (*P* < 0.05).

### Expression Analysis in Developmental Stages

During the different developmental stages, *Mn-ferritin* expression was generally higher during the post-larval developmental stages than the larval developmental stages. *Mn-ferritin* expression remained stable during the larval developmental stages, with no significant difference in expression levels (*P* > 0.05). *Mn-ferritin* expression peaked during the post-larval developmental stage (PL) at PL5 (*P* < 0.05) and remained at a high level until PL15 (Figure 1B). *Mn-ferritin* expression level in the testis of the non-reproductive season was 2.43-fold higher than that in the testis of the reproductive season (*P* < 0.05) (Figure 1C).

### *In situ* Hybridization Analysis

**FIGURE 2:**
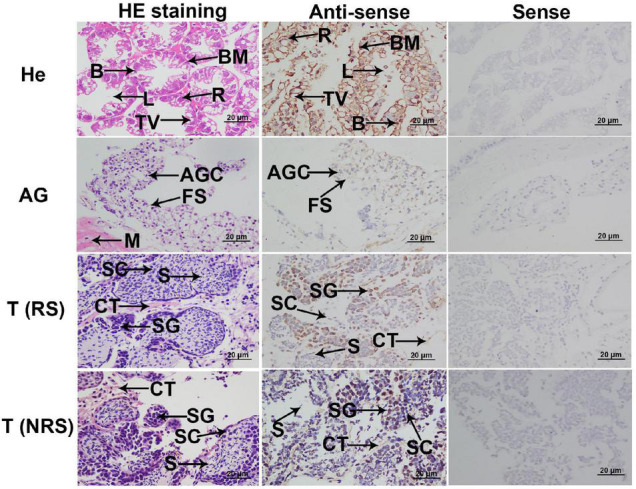
*In situ* hybridization analysis of ferritin in *M. nipponense*. *In situ* hybridization analyses of ferritin were performed in hepatopancreas, testis, and androgenic gland. AG, androgenic gland; AGC, androgenic gland cells; B, secretory cells of type B; BM, basement membrane; CT, connective tissue; FS, funicular structure; He, hepatopancreas; L, lumen; M, muscle; R, storage cells of type R; T (NRS), testis of the non-reproductive season; T (RS), testis of the reproductive season; TV, transferred vacuoles; S, sperm; SC, spermatocyte; SG, spermatogonia. Scale bars = 20 μm. The positively stained cells with DIG signals in antisense labels are characterized by “brown color”. Strong DIG signals were observed in all cell types of the hepatopancreas. Strong DIG signal was only observed in the spermatogonia in the testis of the non-reproductive season and reproductive season. Strong DIG signal was also observed in the funicular structure, surrounding the androgenic gland cells.

*In situ* hybridization was used to determine the mRNA locations of *Mn-Ferritin* in different tissues. According to the HE staining, the cell types in hepatopancreas were secretory cells, basement membrane, lumen, storage cells and transferred vacuoles, and strong DIG signals were also observed in these cells (Figure 2). Sperm was the dominant cell type in the testis of the reproductive season, as well as a small number of spermatogonia and spermatocytes. However, the dominant cell types in the testis of the non-reproductive season were spermatogonia and spermatocytes. The DIG signals in the testis of the non-reproductive season were stronger than that in the reproductive season. Androgenic gland cells and funicular structures were observed in the androgenic gland (Figure 2). Strong DIG signals for *Mn-ferritin* were only observed in spermatogonia in the testis of *M*. *nipponense*, revealed by *in situ* hybridization analysis. No DIG signals were observed in spermatocytes and sperm. In the androgenic gland, no DIG signal was directly observed in the androgenic gland cells. In addition, strong DIG signals were observed in the funicular structure surrounding the androgenic gland cells (Figure 2). No DIG signals were observed when the sense RNA probe was used.

### RNA Interference Analysis

**FIGURE 3:**
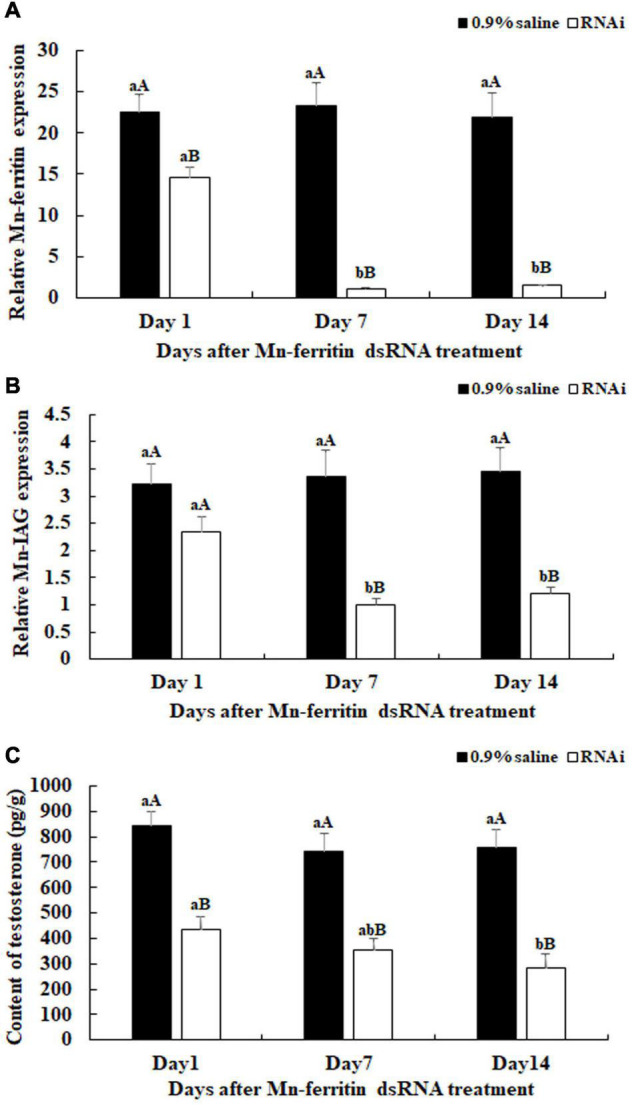
Expression characterization of *Mn-ferritin* and *Mn-IAG*, and measurement of the contents of testosterone at different days after *Mn-ferritin* dsRNA injection. The amount of *Mn-ferritin* and *Mn-IAG* mRNA was normalized to the *EIF* transcript level. Data are shown as mean ± SD (standard deviation) of tissues from five biological replicates. Lowercases indicated significant expression difference among different time points in the same treated group and capital letters indicated the significant difference between the RNAi group and control group on the same day after treatment (*P* < 0.05). The *Mn-ferritin* expressions, *Mn-IAG* expressions, and the contents of testosterone showed slight differences on different days after the injection of 0.9% saline (*P* > 0.05). However, the *Mn-ferritin* expressions, *Mn-IAG* expressions, and the contents of testosterone were significantly decreased on different days after the injection of *Mn-ferritin* dsRNA, and showed significant differences on days 7 and 14 (*P* < 0.01), compared to those of 0.9% saline injection. **(A)** Expression characterization of *Mn-ferritin* after *Mn-ferritin* dsRNA injection. **(B)** Expression characterization of *Mn-IAG* after *Mn- ferritin* dsRNA injection. **(C)** Measurement of the contents of testosterone after *Mn-ferritin* dsRNA injection.

This study aimed to analyze the potentially novel functions of *Mn-ferritin* during sexual development in male *M*. *nipponense* using RNAi. Male prawns were used for RNAi analysis. qPCR analysis revealed that *Mn-ferritin* expression remained at a stable level on different days after the treatment of 0.9% saline (*P* > 0.05). However, *Mn-ferritin* expressions were dramatically decreased after the treatment with *Mn-ferritin* dsRNA. *Mn-ferritin* expressions decreased over 90% at day 7 and day 14 after the *Mn-ferritin* dsRNA injection, compared with that of the 0.9% saline injection on the same day (*P* < 0.01) (Figure 3A).

*Mn-IAG* expression was measured using the same cDNA template after the injection of *Mn-ferritin* dsRNA. The qPCR analysis revealed that there were no significant differences in *Mn-IAG* expression on different days after the injection of 0.9% saline (*P* > 0.05). However, the *Mn-IAG* expressions in the RNAi group decreased with decreasing *Mn-Ferritin*. The *Mn-IAG* expression on day 1 in the RNAi group only decreased by 25%, compared with the injection of 0.9% saline on the same day. However, expression levels decreased by over 60% on days 7 and 14 and showed significant differences with the injection of 0.9% saline on the same day (*P* < 0.01) (Figure 3B).

Testosterone levels were measured on days 1, 7, and 14 after *Mn-ferritin* dsRNA injection. Testosterone levels significantly decreased (up to 60%) on days 7 and 14 in the RNAi group, compared with that of 0.9% saline injection on the same day (*P* < 0.01) (Figure 3C).

### Histological Observations

**FIGURE 4:**
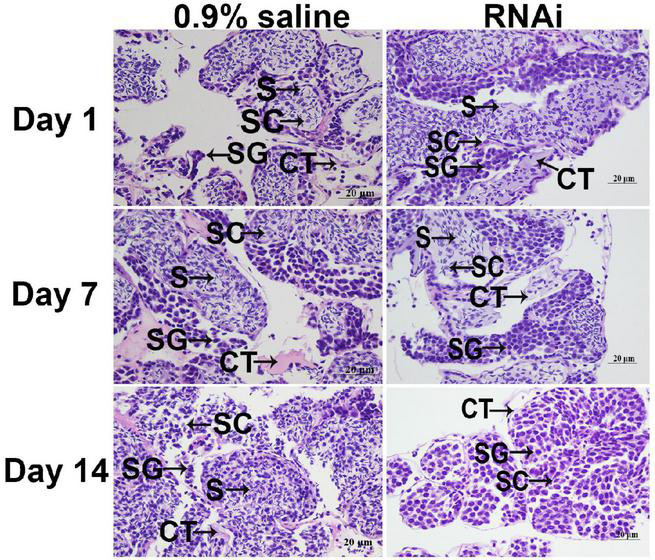
Histological observations of testis between 0.9% saline and *Mn-ferritin* dsRNA treated group by HE staining. Sperm was the dominant cell type in the testis of 0.9% saline group, while the number of sperm gradually decreased from day 1 to 14 after the *Mn-ferritin* dsRNA injection. CT, connective tissue; S, sperm; SC, spermatocyte; SG, Spermatogonia. Scale bars = 20 μm.

There were no significant differences in the testis on different days in the control group. Sperm cells were the dominant cell type in the testis of the 0.9% saline injection. However, the number of sperm gradually decreased from day 1 to 14 after the *Mn-ferritin* dsRNA injection. The numbers of spermatogonia and spermatocytes were much higher than the number of sperm on day 7 in the RNAi group, and sperm was rarely recorded on day 14 in the RNAi group (Figure 4).

## Discussion

Ferritin has been confirmed to be involved in multiple physiological function regulation, such as development, cell activation, and angiogenesis (Parthasarathy et al., 2002; Coffman et al., 2008; Alkhateeb and Connor, 2010; Wang et al., 2010). Recent studies showed that it was differentially expressed in the proteomic profiling analysis of the androgenic gland between *M*. *nipponense* in reproductive season and non-reproductive season (Jin et al., 2018a). In addition, ferritin also showed a higher expression level in the androgenic gland than that of the testis and ovaries, verified by qPCR (Jin et al., 2018a). Thus, ferritin was predicted to be involved in the mechanism of male sexual development in *M*. *nipponense*, due to the positive regulatory role of the androgenic gland in male sexual development in crustacean species (Jin et al., 2018a). In the present study, we aimed to further investigate the potentially novel functions of ferritin in the mechanism of male sexual development of *M*. *nipponense*.

The tissue distributions of ferritin showed different expression patterns in various species. Ferritin exhibited the highest expression in the hemocytes of *L. vannamei* (Hsieh et al., 2006), while *Pacifastacus leniusculus* had the highest expression levels of ferritin in the hepatopancreas (Huang et al., 1996). In the current study, ferritin also showed the highest expression level in the hepatopancreas of *M. nipponense*, followed by the testis and the androgenic gland, whereas expression in the ovary was relatively low. Similar results were also obtained in *M. rosenbergii* (Qiu et al., 2008). However, the prawn hepatopancreas was not responsive to iron injections. Thus, the high expression of ferritin in the hepatopancreas could be regulated at the post-transcriptional level (Huang et al., 1999). Previous studies revealed that ferritin also showed the highest expression levels in the hepatopancreas, and the levels were upregulated after iron and pathogen challenge in *M. nipponense*, indicating that ferritin played essential roles in organismic and cellular iron homeostasis of *M. nipponense*, protecting the cells from damage by excess iron (Sun et al., 2014). In addition, the expressions of *Mn-ferritin* were higher in the testis and the androgenic gland than the other tested tissues (*P* < 0.05), except hepatopancreas in this study. This indicates that ferritin may have additional functions in the mechanism of male sexual development in *M. nipponense*, based on the essential roles of the testis and the androgenic gland on male sexual development in crustacean species. Furthermore, this indicates that iron homeostasis may mediate gonad development in *M. nipponense*. The *Mn-ferritin* expression in the testis was slightly higher than that in the androgenic gland in this study (*P >* 0.05), which is different from the previous study. A reasonable explanation for the difference is that the testis development is continuous. Thus, the cell numbers of spermatogonium, spermatocyte, and sperm in the testis of this study may be different from those in the previous study.

The ferritin expression has shown dramatic differences during different developmental stages of aquatic species. During continuous developmental stages, ferritin was expressed in the zoea stage, as well as the megalopa and juvenile crab I stage in *Scylla paramamosain* (Zhang et al., 2011). The ferritin of *Exopalaemon carinicauda* was rarely expressed at the gastrula and zoea stage, whereas it was significantly upregulated from the egg protozoea stage, and showed the highest expression level at the post-larvae stages (Zhang et al., 2015). In the present study, the expression level of ferritin remained stable and low during the larval developmental stages of *M. nipponense*, compared to those of the post-larval developmental stages. During the post-larval developmental stages, *Mn-ferritin* expression increased dramatically, peaked at PL5, and remained at a high level until PL15. *Mn-ferritin* exhibited higher expression levels during the post-larval developmental stages than the larval developmental stages. Histological observations during the larval and post-larval developmental stages of *M*. *nipponense* have proven that the sex-differentiation sensitive period of *M*. *nipponense* is from PL7 to PL22 (Jin et al., 2016), during which time the androgenic gland, testis, and ovary differentiated and matured. The dramatically high expression levels from PL5 to PL15 indicated that ferritin may play vital roles in gonad differentiation and development in *M*. *nipponense*.

The *in situ* hybridization analysis of ferritin has been reported for several species. A ferritin homolog is ubiquitously expressed in *Branchiostoma belcheri* (Li et al., 2008). Ferritin mRNA is highly expressed in the mantle fold of *Pinctada fucata* (Zhang et al., 2003). L-ferritin mRNA was observed to be upregulated in iron-loaded rats. L-ferritin mRNA was localized in the colonic crypt, villus epithelial cells, small intestinal crypt, and surface epithelial cells (Jeffrey et al., 1996). In the present study, strong DIG signals for ferritin were observed in all cell types in the hepatopancreas, indicating that the hepatopancreas was the main organ for iron homeostasis in *M. nipponense*. Interestingly, strong DIG signals were only detected in spermatogonia in the testis of both reproductive season and non-reproductive season, indicating that ferritin may promote spermatogonia development or activate testis development. DIG signals in the testes from the non-reproductive season were stronger than those from the reproductive season. qPCR analysis revealed that *Mn-ferritin* in the testes of the non-reproductive season was higher than in those of the reproductive season, which is consistent with the *in situ* hybridization analysis. Histological observations revealed that the main cell type in the testis of the reproductive season was sperm, while spermatogonia and spermatocytes were the dominant cell type in the testis of the non-reproductive season (Jin et al., 2020). This result further confirmed that ferritin promoted spermatogonia development. Our data also showed that strong DIG signals were detected in the funicular structure surrounding the androgenic gland cells, while no signal was directly detected in the androgenic gland cells. Previous research indicated that the androgenic gland development began with the formation of the funicular structure, then androgenic gland cells were formed and filled the funicular structure (Jin et al., 2016). The *in situ* hybridization of ferritin in the androgenic gland of *M. nipponense* suggested ferritin played essential roles in the development of the funicular structure, most likely supporting the formation of androgenic gland cells.

RNA interference was efficiently used to analyze the gene functions through knockdown of the gene expression of target genes, which has been widely used in *M*. *nipponense* (Jiang et al., 2014; Li et al., 2015a,^2018^). In this study, *Mn-ferritin* expression levels on days 7 and 14 in the RNAi group were significantly lower than the control group on the same day, indicating the dsRNA can efficiently inhibit *Mn-ferritin* expression. We also found that, after the injection of *Mn-ferritin* dsRNA, the *Mn-IAG* expressions decreased with decreasing *Mn-ferritin* expression, indicating that ferritin positively regulated *IAG* expression in *M. nipponense*. *IAG* has been reported to positively promote the male differentiation and development in crustacean species (Ventura et al., 2009, 2011). RNAi of *IAG* showed a significant inhibitory effect on male sexual differentiation and development of secondary sexual characteristics, including the formation of spermatogenesis in *M. rosenbergii* (Ventura et al., 2012). *IAG* has been proven to be expressed in the androgenic gland of *M. nipponense* (Ma et al., 2016). Furthermore, the expressions of *IAG* were regulated by the expressions of crustacean hyperglycemic hormone, gonad inhibiting hormone, and molting inhibiting hormone in *M. nipponense* (Li et al., 2015b). Our data also revealed that the testosterone levels in the RNAi group were lower than that of control group on the same day. Testosterone has been proven to result in sex reversal in *Oreochromis mossambicus* (Cao et al., 1994), allogynogenetic crucian carp (*Carassius auratus*) (Lou et al., 1994; Zhang et al., 2000), *Xiphophorus hellerii* (Han et al., 2010) and the grouper *Epinephelus akaara* (Li et al., 2006). In addition, testosterone promotes the development of testes and sperm in *Eriocheir sinensis*, and is involved in early gonad differentiation and development in *M*. *nipponense* (Jin et al., 2016). The positive regulatory roles between the expression of ferritin and *IAG*, and the secretion of testosterone indicated that ferritin was involved in the mechanism of male sexual development in *M. nipponense*. Histological observations of testis indicated that the number of sperm decreased dramatically after injection of *Mn-ferritin* dsRNA. Sperm was rarely found on day 14 after the injection of *Mn-ferritin* dsRNA. This indicates that ferritin was involved in the regulation of testis development in *M. nipponense* by inhibiting the expression of *Mn-IAG* and the secretion of testosterone, indicating iron homeostasis was involved in the process of testis development in *M. nipponense*.

## Conclusion

We proved that ferritin played an essential role in the sexual development of male *M. nipponense*. Ferritin exhibited a positive regulation in *M. nipponense* testis development by regulating *Mn-IAG* expression and the secretion of testosterone, indicating that iron homeostasis was also involved in the testis development of *M. nipponense*. The present study provided an essential basis for the future establishment of novel techniques to regulate testis development in *M*. *nipponense*.

## Data Availability Statement

The original contributions presented in the study are included in the article/supplementary material, further inquiries can be directed to the corresponding author/s.

## Ethics Statement

The animal study was reviewed and approved by the Institutional Animal Care and Use Ethics Committee of the Freshwater Fisheries Research Center, Chinese Academy of Fishery Sciences (Wuxi, China). Written informed consent was obtained from the owners for the participation of their animals in this study.

## Author Contributions

SBJ designed and wrote the manuscript. HF supervised the experiment. SFJ provided the experimental prawns. YX performed the qPCR analysis. HQ performed the *in situ* hybridization analysis. WZ performed the RNAi analysis. YG performed the histological observations. YW revised the manuscript. All authors contributed to the article and approved the submitted version.

## Conflict of Interest

The authors declare that the research was conducted in the absence of any commercial or financial relationships that could be construed as a potential conflict of interest.

## Publisher’s Note

All claims expressed in this article are solely those of the authors and do not necessarily represent those of their affiliated organizations, or those of the publisher, the editors and the reviewers. Any product that may be evaluated in this article, or claim that may be made by its manufacturer, is not guaranteed or endorsed by the publisher.

## Footnotes

1
https://www.flyrnai.org/cgi-bin/RNAi_find_primers.pl
